# Evaluation of methods for oligonucleotide array data via quantitative real-time PCR

**DOI:** 10.1186/1471-2105-7-23

**Published:** 2006-01-17

**Authors:** Li-Xuan Qin, Richard P Beyer, Francesca N Hudson, Nancy J Linford, Daryl E Morris, Kathleen F Kerr

**Affiliations:** 1 Department of Epidemiology and Biostatistics, Memorial Sloan-Kettering Cancer Center, New York, New York, USA; 2 Department of Environmental Health, University of Washington, Box 354695, Seattle, Washington, USA; 3 Department of Pathology, University of Washington, Box 357705, Seattle, Washington, USA; 4 Department of Biostatistics, University of Washington, Box 357232, Seattle, Washington, USA

## Abstract

**Background:**

There are currently many different methods for processing and summarizing probe-level data from Affymetrix oligonucleotide arrays. It is of great interest to validate these methods and identify those that are most effective. There is no single best way to do this validation, and a variety of approaches is needed. Moreover, gene expression data are collected to answer a variety of scientific questions, and the same method may not be best for all questions. Only a handful of validation studies have been done so far, most of which rely on spike-in datasets and focus on the question of detecting differential expression. Here we seek methods that excel at estimating relative expression. We evaluate methods by identifying those that give the strongest linear association between expression measurements by array and the "gold-standard" assay.

Quantitative reverse-transcription polymerase chain reaction (qRT-PCR) is generally considered the "gold-standard" assay for measuring gene expression by biologists and is often used to confirm findings from microarray data. Here we use qRT-PCR measurements to validate methods for the components of processing oligo array data: background adjustment, normalization, mismatch adjustment, and probeset summary. An advantage of our approach over spike-in studies is that methods are validated on a real dataset that was collected to address a scientific question.

**Results:**

We initially identify three of six popular methods that consistently produced the best agreement between oligo array and RT-PCR data for medium- and high-intensity genes. The three methods are generally known as MAS5, gcRMA, and the dChip mismatch mode. For medium- and high-intensity genes, we identified use of data from mismatch probes (as in MAS5 and dChip mismatch) and a sequence-based method of background adjustment (as in gcRMA) as the most important factors in methods' performances. However, we found poor reliability for methods using mismatch probes for low-intensity genes, which is in agreement with previous studies.

**Conclusion:**

We advocate use of sequence-based background adjustment in lieu of mismatch adjustment to achieve the best results across the intensity spectrum. No method of normalization or probeset summary showed any consistent advantages.

## Background

Affymetrix GeneChip^® ^oligonucleotide arrays are a popular platform for the high-throughput analysis of gene expression in mRNA. Nguyen et al [1] give an introduction to the technology for quantitative scientists. Briefly, an oligonucleotide array contains 11–20 probe pairs for each gene. Probe pairs consist of an oligonucleotide that is a "perfect match" (PM) to a subsequence of the mRNA transcript for a gene and a corresponding "mismatch" (MM) oligo that differs from it in one base in the middle. These MM probes are meant to provide information on cross-hybridization.

Quantitative researchers have proposed a variety of methods for handling probe-level data from Affymetrix^® ^oligonucleotide arrays. Methods employ different procedures for adjusting for background fluorescence, normalizing the data, incorporating the information from "mismatch" probes, and summarizing probesets (combining all the data from the different probes for a given gene). In particular, the value and proper use of data from MM probes have been subjects of some controversy [2]. It is important to validate a method for its effectiveness in achieving scientific goals, such as estimating relative gene expression or detecting differentially expressed genes [3]. Note that different methods may be preferable for different scientific goals [4].

Previously, spike-in studies have been used to study the variance and bias of different estimates of relative expression derived from oligo array data. These studies are useful and important, but are not the end of the story. First, spike-in datasets are inherently artificial, and may not realistically represent the operating characteristics of a methodology on real data [5]. For example, the Affymetrix Latin Square Dataset studied by Bolstad et al [6] has only 42 genes changing from sample to sample. In addition, this dataset was used to develop several methods, so it is not appropriate to use for validation. Finally, a criterion often not considered in the spike-in studies is the accuracy of measurements *across genes*. Instead, Bolstad et al [6] largely considered measurements across RNA samples for single genes. Obviously, these problems are related, yet they are not identical.

Choe et al [7] conducted a study using an experiment where 100–200 RNAs were spiked-in at various fold-changes. All RNAs other than the spike-ins had the same level in all samples. Impressively, the authors considered over 100 different combinations of methods for background adjustment, normalization, use of MM probes, and probeset summary methods. Many of the study's conclusions are based on the shared features of the ten best-performing combinations. However, eight of those ten combinations used a normalization based on the known subset of genes that were constant between the RNAs that were compared. Such a normalization scheme could not be implemented in an actual experiment where the identity of unchanging genes is unknown. This casts some doubt on the generalizability of the study's findings. Further concerns about generalizability arise from the study's non-standard RNA production protocol. In addition, one of the study's RNA samples contained unlabeled poly(C) RNA, to unknown effect.

Among evaluations that do not rely on spike-in datasets, Ploner et al [8] favored methods that produced zero correlation, on average, between randomly selected pairs of genes. Though creative, this criterion unfortunately does not correspond to a scientific question of interest. Furthermore, the criterion might favor methods that "over-normalized" the data – removed signal as well as systematic biases. Shedden et al [5] identified methods that optimized sensitivity for detecting differentially expressed genes. The authors relied on estimates of false discovery rates rather than using data from an independent validation technique for comparison.

In contrast to the studies that use spike-in datasets, our study is based on a real dataset that was collected to answer biological questions. The studies by Choe et al [7] and Shedden et al [5] are directed at identifying the best methods for selecting differentially expressed genes, which are not necessarily the best methods for estimating relative expression. In contrast, we focus here on the problem of estimating relative expression. We do not mean to suggest that previous approaches lack merit. Rather, different approaches have advantages and disadvantages, and a plurality of studies is needed.

**Figure 1:**
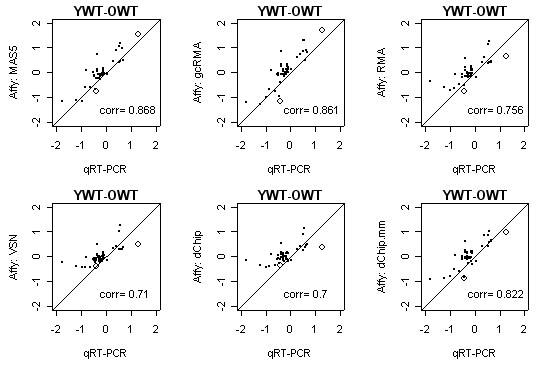
**Agreement between array and qRT-PCR for the comparison of Y-WT and O-WT mice. **For the Y-WT vs. O-WT contrast, the figure shows estimates of relative expression from the array data, processed with six different methodologies, compared to qRT-PCR. Estimated differences are on the log_2 _scale. Genes indicated with an open circle are influential genes according to the sensitivity analysis. The number on each scatterplot is the Pearson correlation.

In our experiment, heart tissue was collected from 24 individual mice in a 2 × 2 design (see Table 1). Affymetrix GeneChips^® ^(Murine Genome Array U74Av2) were used to assay RNAs from these tissue samples. Quantitative RT-PCR measurements for 47 genes were taken on these same 24 RNAs. As the "gold-standard" method of measuring gene expression, we treat the qRT-PCR measurements as "truth" for the purposes of this study. In our "overview" investigation, array data were processed in six different ways to arrive at estimates of gene expression among the 24 mice. The six methodologies are MAS5, gcRMA, RMA, VSN, and two versions of dChip (see Table 2 for information on methods and references). We used Pearson Correlation to measure the agreement between array and qRT-PCR measurements on six group comparisons, or "contrasts," among the mice (see the 'Contrasts' section of METHODS and Figure 1). In a follow-up investigation, we considered 56 different combinations of the components of these methods.

Our choice to use Pearson's correlation, r, is motivated by the following formula. While not the standard textbook definition of r, a more instructive approximate formula is

r≈β2Var(X)β2Var(X)+Var(Y|X)
 MathType@MTEF@5@5@+=feaafiart1ev1aaatCvAUfKttLearuWrP9MDH5MBPbIqV92AaeXatLxBI9gBaebbnrfifHhDYfgasaacH8akY=wiFfYdH8Gipec8Eeeu0xXdbba9frFj0=OqFfea0dXdd9vqai=hGuQ8kuc9pgc9s8qqaq=dirpe0xb9q8qiLsFr0=vr0=vr0dc8meaabaqaciaacaGaaeqabaqabeGadaaakeaacqWGYbGCcqGHijYUdaGcaaqaamaalaaabaGaeqOSdi2aaWbaaSqabeaacqaIYaGmaaGccqWGwbGvcqWGHbqycqWGYbGCcqGGOaakcqWGybawcqGGPaqkaeaacqaHYoGydaahaaWcbeqaaiabikdaYaaakiabdAfawjabdggaHjabdkhaYjabcIcaOiabdIfayjabcMcaPiabgUcaRiabdAfawjabdggaHjabdkhaYjabcIcaOiabdMfazjabcYha8jabdIfayjabcMcaPaaaaSqabaaaaa@4DAE@

where β is the slope of the line for predicting Y from X, Var(X) is the variance of X, and Var(Y|X = x) is the variance of Y in groups that have the same value of X. In our application, X is the measurements from qRT-PCR and Y is the measurements from array. X is fixed, and so also Var(X), but Y depends on what method is applied to the array data. Since Var(Y|X = x) appears in the denominator, a method's performance is improved if it minimizes Var(Y|X = x). Therefore, this metric tends to favor methods with smaller variability. Similarly, the larger the slope between Y and X, the larger r is and the more favorable a method's performance. In this sense, by using Pearson's correlation, we simultaneously take into account both the variance and bias of the measurements produced by arrays. That is, we seek methods that achieve the right balance between variance and bias to yield the strongest association between array measurements and qRT-PCR. However, it is also of interest to specifically examine variance and bias, and we will come back to this.

**Figure 2:**
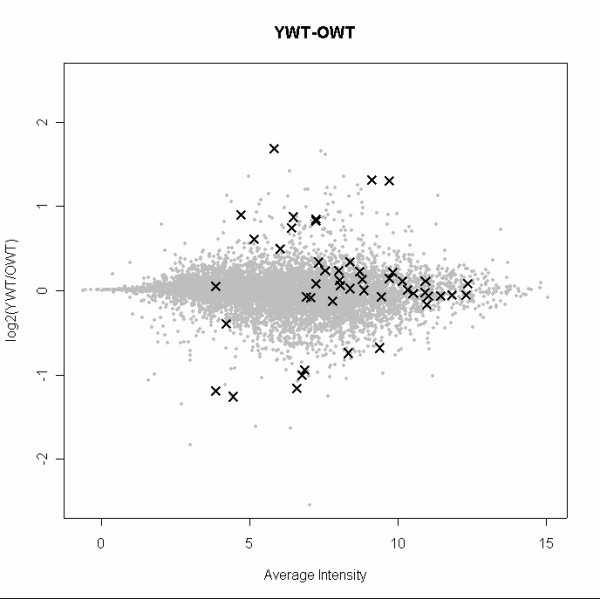
**Genes selected for qRT-PCR are medium to high intensity in array data. **The plot highlights the genes selected for qRT-PCR in a scatterplot of the YWT vs. OWT contrast against the mean signal intensity. Data were processed with gcRMA for this plot. Selected genes span a large range of average signal intensity with the notable exception of low-intensity genes. See AdditionalFigures.doc for similar figures for the other contrasts.

We note that the 47 genes assayed with qRT-PCR were selected based on primer availability, initial evidence for differential expression, signal intensity, and biological interest. The 47 genes do not comprise a random sample. In particular, the genes for which we have qRT-PCR data do not include low-intensity genes (see Figure 2 and AdditionalFigures.doc for the additional contrasts). The 47 genes are medium- and high-intensity genes, with the larger fold-changes tending to be for the medium-intensity genes. Therefore, our results about inter-platform agreement pertain primarily to high- and especially medium-intensity genes. We will return to this important issue.

## Results

### MAS5, gcRMA, and dChip mismatch model achieve the best agreement between array and qRT-PCR

**Figure 3:**
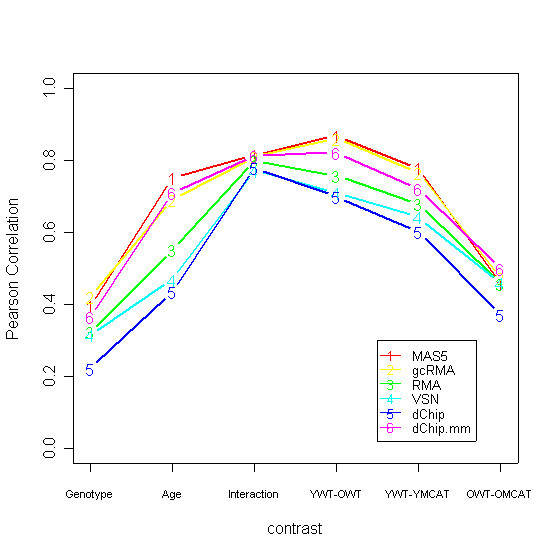
**Relative performance of the six methodologies for six summary contrasts of the data. **MAS5, gcRMA, and dChip.mm consistently outperform the other methods, although all methods performed comparably on the 'Interaction' contrast. Correlations are lower for contrasts for which there is less differential expression, as seen in the scatterplots such as Figure 1 [see AdditionalFigures.doc]. However, the interesting comparisons are between the six correlations for a given contrast.

We examined six methods (see Table 2) to identify those that yield the strongest linear association between array and qRT-PCR measurements of relative gene expression (see METHODS). Figure 3 shows that three methods, MAS5, gcRMA, and dChip.mm, consistently outperform the other three. While we are not able to compute confidence intervals to evaluate the statistical significance of the differences, we argue that the improvement in correlation by using one of the three best methods is compelling. Some caution is warranted however, due to the non-random selection of the genes; see DISCUSSION.

We conducted sensitivity analyses out of concern that a single gene or a pair of genes might disproportionately influence the results, which is a general concern with the correlation metric. Table 3 gives the results of the leave-one-out sensitivity analysis. Across the six contrasts, there were 41 instances where the ranking of methodologies changed when one gene was left out of the dataset. However, in 39 of these instances the ranking changed via a transposition of two adjacent methodologies in the rankings, or a shuffle of three adjacent methodologies. The only exceptions are (1) the interaction contrast and gene 12, and (2) the OWT-OMCAT contrast and gene 26. Removing gene 12 produces a shuffle of the top four performing methodologies for the interaction contrast. Removing gene 26 produces a shuffle of the top five performing methodologies for the OWT-OMCAT contrast. Notice, however, that the rankings are inherently unstable for the interaction contrast simply because all methodologies performed comparably for this contrast (Figure 3). Similarly, the top five methodologies performed comparably for the OWT-OMCAT contrast, so a shuffle among them is not alarming.

Table 4 summarizes the results for the leave-two-out sensitivity analysis. In total, these sensitivity analyses provide assurance that our results are robust and not overly influenced by a single or pair of genes in our study.

### Variability is intensity-dependent

**Figure 4:**
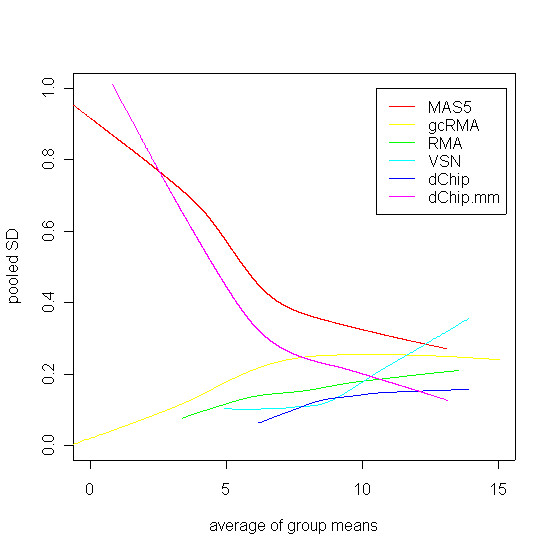
**Variability of methods within biological replicates as related to signal intensity. **For each version of the data, the standard deviation of measurements within each of the four biological groups was calculated, and these were pooled to form a single standard deviation for each gene. These were plotted against the mean intensity for that gene, and fitted with a non-parametric smoother to summarize the trend. The fitted smooths are shown above. MAS5 shows the greatest variability, and MAS5 and the dChip mismatch model both show greater variability at low intensities.

Figure 4 shows the variance of methods within biological replicates. We see that variability is highest for MAS5. MAS5 and the dChip mismatch model exhibit dramatically increased variability at lower intensities. This increased variability could be explained by greater biological variability at low intensities. However, statistically, one expects that subtracting mismatch data should increase variability, and that excess variability would be dramatic at low intensities [9]. This explanation is also entirely consistent with previous empirical data [2]. The four methods that do not use MM probe data are roughly comparable across the intensity spectrum with respect to variability, with the dChip method exhibiting the smallest variability. An interesting side note is that VSN does not exhibit constant variability across the intensity spectrum, despite incorporating a transformation specifically intended to achieve this.

### For medium- and high-intensity genes, variability is offset by reduced bias

**Figure 5:**
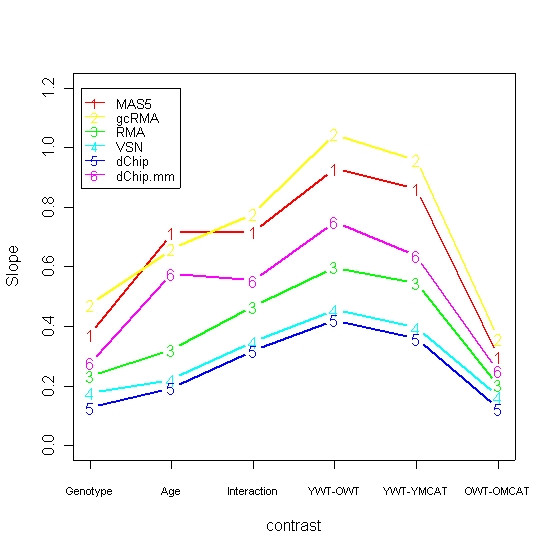
**Bias across contrasts for the six methods. **Slopes of the least squares regression lines fitted to scatterplots such as in Figure 1. The three methods that showed the best correlation between array and qRT-PCR, MAS5 gcRMA, and the dChip mismatch model, consistently show the least bias, with slopes closest 1.

Figure 5 is similar to Figure 3 but displays the slope of the least-squares regression lines that we fit to the scatterplots like Figure 1 (data not shown). These slopes can be considered a measure of the bias of methods, with slopes closer to 1 indicating less bias. Notice that the slopes for the three methods with the best correlation, MAS5, gcRMA, and the dChip mismatch model, are consistently closer to 1, and also larger than the slopes for the remaining three methods. Although these methods have more variability (Figure 4), they achieve better agreement with qRT-PCR by having smaller bias.

### No apparent relationship between gene characteristics and agreement between platforms

As an exploratory aspect of our study, we sought to identify factors that influence the level of agreement between array and qRT-PCR measurements. We considered whether gene sequence GC content or Affy probe GC content was associated with agreement in the measurements produced by the two platforms. Neither variable showed a consistent association. See ProbeLevelAnalysis.doc for more information on this exploratory analysis.

We also failed to corroborate the finding of Etienne et al [10] that large distance between qRT-PCR probe and the Affy probe set leads to poor agreement between platforms. We believe the likely reason for this discrepancy is the difference in RT-PCR methods. Etienne et al [10] used standard 2 primer PCR followed by radioactive Southern blot hybridization. The use of a real time PCR machine in our study allowed greater assurances that all amplifications measured were consistent, specific, and within the appropriate linear range. Our use of the Taqman system with a fluorogenic minor groove binding probe also increased specificity and stabilized binding sites. These factors combined could reduce any sequence-specific error in qRT-PCR measurement.

### Use of mismatch data or sequence-based background correction is the most influential factor

We sought to better understand why MAS5, gcRMA, and the dChip mismatch model performed better in our correlation analysis. Similar to Choe et al [7], we considered all compatible combinations of the components that comprise these three methods. Table 5 delineates those methods and also gives a shorthand notation that we will use to discuss them here. Altogether, we evaluated 56 combinations.

**Figure 6:**
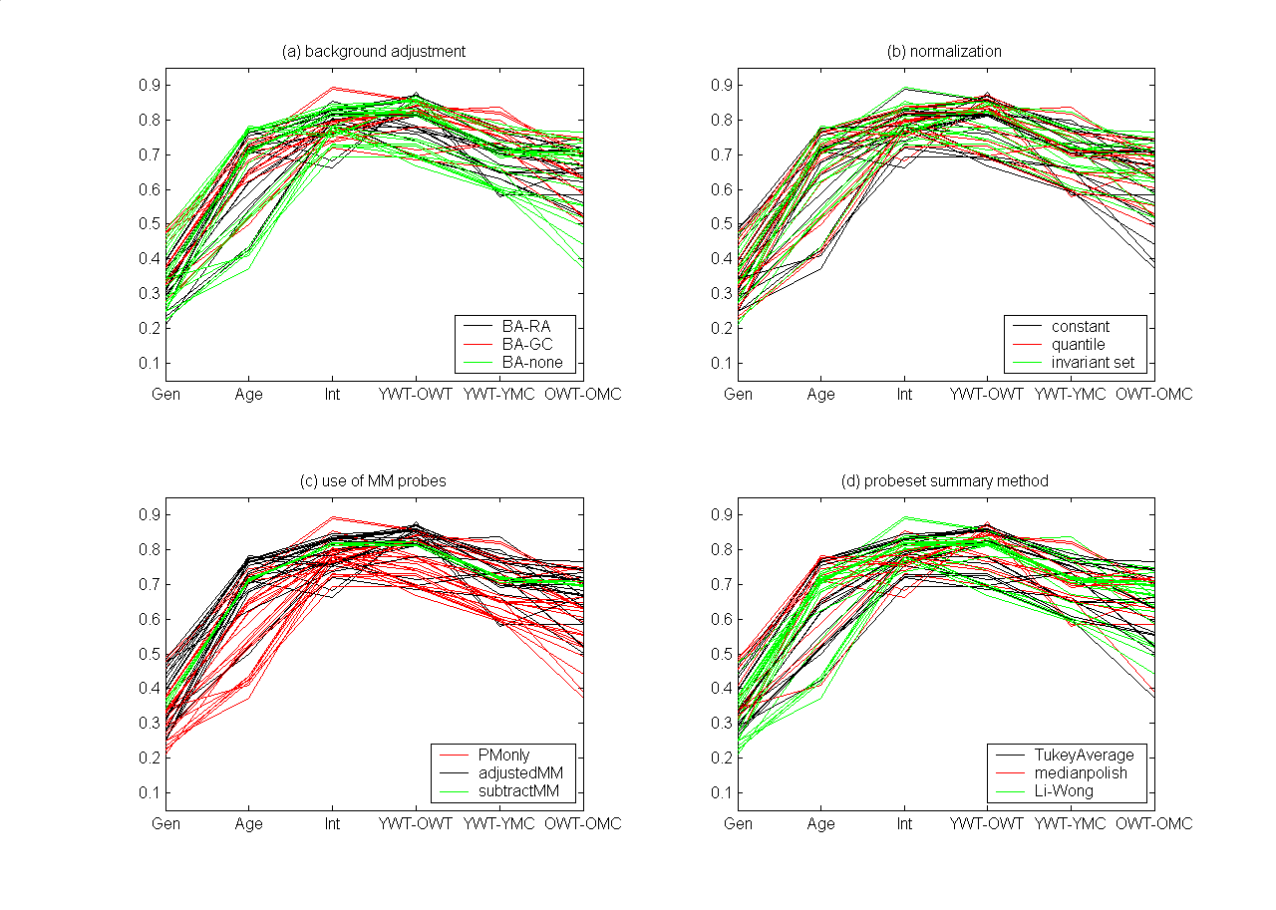
**Correlations for each of the six contrasts for 56 combinations. **See Table 5 for notation. Curves are colored by (a) the method of background adjustment, (b) the method of normalization, (c) the use of MM probe data, (d) the method for summarizing data across a probeset. No sub-method is clearly uniformly superior.

Figure 6 shows a first look at the results of the follow-up analysis. No particular sub-method stands out as uniformly superior to its alternatives. However, this way of viewing results can hide combinations of sub-methods that work well together. For example, we found that each probeset summary method worked well when combined with certain other components.

**Figure 7:**
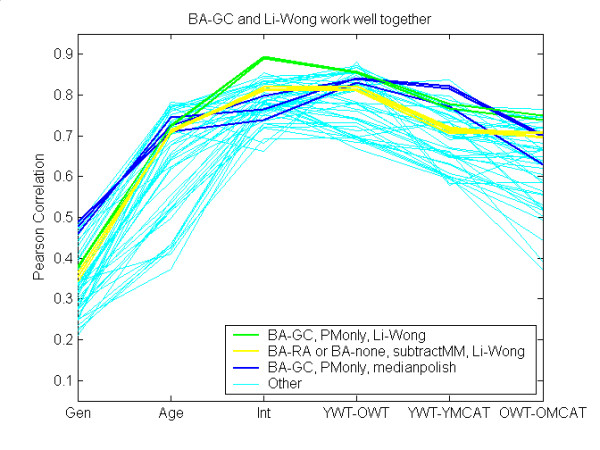
**BA-GC and Li-Wong perform well together. **See Table 5 for notation. Combinations that used both BA-GC and the Li-Wong summary method perform consistently well (green curves). Replacing BA-GC with subtractMM was not as good (yellow curves). Replacing the Li-Wong with medianpolish is also arguably less effective (blue curves). Note: BA-GC could not be combined with subtractMM, because this resulted in negative values.

Using the shorthand notation established in Table 5, Figure 7 shows that the BA-GC and Li-Wong appear to work consistently well together (green curves). Such combinations arguably work better than BA-GC combined with medianpolish (blue curves), even though BA-GC was developed in conjunction with medianpolish. Similarly, combining Li-Wong with subtractMM (yellow curves) instead of BA-GC does not correlate with RT-PCR data quite as well. In other words, combinations of components of two top-performing methods, gcRMA and the dChip mismatch model, outperformed both.

**Figure 8:**
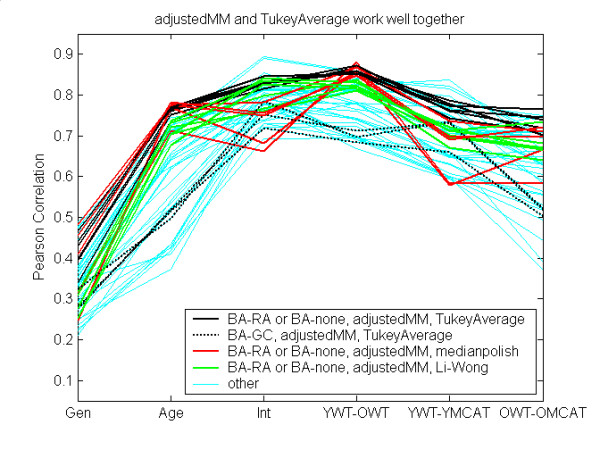
**adjustedMM and TukeyAverage perform well together. **See Table 5 for notation. Combinations that used two components of MAS5, adjustedMM and TukeyAverage, perform consistently well (black solid curves), as long as BA-GC was not also used (dotted black curves). Using another probeset summary method with adjustedMM was not as effective (red and green curves).

As another example, the adjustedMM worked consistently well when combined with TukeyAverage (Figure 8, black curves) as long as BA-GC is not also used (presumably the combination is an over-adjustment). Both adjustedMM and TukeyAverage are components of MAS5. The green and red curves in Figure 8 show the arguably worsened results when adjustedMM was combined with Li-Wong or medianpolish.

**Figure 9:**
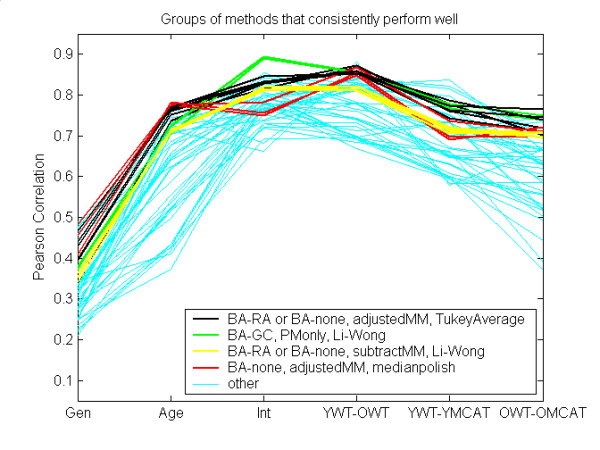
**Groups of methods that consistently performed well. **Methods that combine BA-GC with Li-Wong are consistently near the top (green curves), as well as adjustedMM combined with Tukey Average (black curves), as long as GA-GC was not also used. Other consistent performers are also noted in the Figure.

Figure 9 summarizes results for some groups of methods that consistently performed well. Note that each method of background adjustment, each method for MM probes, and each probeset summary method is involved in at least one group. The most notable feature, however, is that each group in Figure 9 uses exactly one of BA-GC, adjustedMM, or subtractMM. Therefore, these are the components that lead to the superior performance of MAS5, gcRMA, and the dChip mismatch model in our initial analysis. This result, that the method for MM data is the most important choice in data processing, was also found by [11].

Figures 7, 8, 9 also indicate that differences in normalization had a generally minor effect on results – performance changed little when normalization method varied while all other components were held constant. We do not identify any compelling evidence in favor of any particular method for normalization.

## Discussion

Just as we pointed to limitations of previous studies, it is important to point out limitations of this study. One limitation is that the genes assayed with qRT-PCR were not a random sample or even a representative sample of genes on the array (see METHODS). Genes were initially selected primarily for their biological interest, but then some of these candidates were excluded. Two elements of the selection process are notable. First, genes were selected if they appeared to be promising candidates for differential expression based on processing the data with gcRMA. This introduces a possible bias for gcRMA into our results. Second, genes with average signal intensity less than 2 were excluded. These factors resulted in the selection of primarily high- and medium-intensity genes. Previous work [2] has suggested that difficulties with methods that subtract mismatch data arise for low-intensity genes due to extreme variability. It is likely that the omission of low-intensity genes in our study favored MAS5 and the dChip mismatch model. The remaining criteria used to select genes were the availability of RT-PCR assays and whether existing knowledge of a gene made it an interesting candidate in the study of aging. We are unaware of any biases produced by these latter selection criteria.

We argue that correlation is a reasonable measure of agreement in this study because it accounts for both the bias and variance of measurements, favoring methods that find the right balance between the two. However, Figure 4 shows that "the right balance" really depends on signal intensity. For example, for highly expressed genes, the variability across methods is roughly comparable and so our metric favors methods with the least bias. Figure 3 and Figure 5 show that this is exactly what happens. For genes at the lowest level of intensity, methods that use mismatch probes have been found to be extremely variable [2], which is consistent with our data (Figure 4). For such genes our metric favors methods with lower variability even if the bias is large. Unfortunately, the qRT-PCR analysis did not include low intensity genes. While we had qRT-PCR data for some high-intensity genes, they tended to have smaller fold-changes across group and thus exerted less influence on the correlations (see Figure 2 and similar figures in AdditionalFigures.doc). Our correlation results really pertain to medium-intensity genes, where bias and variation both come into play as sources of error.

Our results, narrowly interpreted, favor MAS5, gcRMA, and the dChip mismatch model. However, our assessment of variability, together with previous studies that demonstrate the unreliability of using MM data for low-intensity genes [2], leads to a more precise conclusion. Specifically, the sequence-based background adjustment of gcRMA emerges as a method that may be most effective across the intensity spectrum.

We have treated the qRT-PCR measurements as "truth" because they are the gold standard laboratory measurement of gene expression. Yet qRT-PCR measurements are also subject to error. However, our sensitivity analysis should partly address this concern.

We reiterate that we have compared the performance of array methodologies for estimating relative gene expression levels for a chosen list of genes. We have not compared methods on their abilities to identify differentially expressed genes, which is an important goal that is related but not identical. Still, it is useful to compare our findings with other validation studies, including those that used other criteria to evaluate methods. Of the three recent studies [5,7,8], our results are somewhat consistent with Choe et al [7] and Ploner et al [8] and least consistent with Shedden et al. [5]. Choe et al [7] concluded that (1) regional background adjustment is better than foregoing background adjustment, (2) using the MAS5 method for use of MM probe data is better than simple MM subtraction or discarding MM data, and (3) the probeset summary method used by gcRMA and RMA performs slightly better than the methods used by MAS5 or the dChip model. Our results suggest a more complicated scenario – that each of these sub-methods performs well if combined with particular other sub-methods. On the other hand, we clearly corroborate the finding of Choe et al [7] that no method of normalization appears to be advantageous, and that gcRMA and MAS5 perform well. Ploner et al [8] concluded that MAS5 gave better results than RMA or the dChip mismatch model. We also found that MAS5 outperformed RMA but it was not clearly better than the dChip mismatch model. The results of Shedden et al [5] favored the dChip method using MM subtraction over MAS5 and gcRMA, while these three performed comparably for the medium- and high-intensity genes in the main part of our study.

## Conclusion

Using qRT-PCR data as an independent measurement tool, we compared the performance of six methodologies for the quantification of gene expression from Affymetrix oligonucleotide arrays. Three methods – MAS5, gcRMA, and the dChip mismatch model – performed better than VSN, dChip without mismatch, and RMA. The factor driving these results was whether a method used mismatch data or, alternatively, a sequence-based background adjustment. Other differences among methods, such as the normalization scheme, made little difference in overall performance. Further analysis of variability lead us to favor the sequence-based background adjustment over procedures using mismatch probes. In summary, for estimating relative expression using oligonucleotide array data, we advocate (1) foregoing methods that use mismatch subtraction and (2) using the sequence-based background adjustment method in gcRMA.

## Methods

### RNA assays

Total RNA was extracted from flash-frozen heart tissue using Trizol (Invitrogen, Carlsbad, CA) extraction followed by cleanup with the RNeasy kit (Qiagen, Valencia, CA). Samples were prepared for Affymetrix arrays using 7 μg total RNA and following the manufacturer's instructions for One Cycle Eukaryotic Target Preparation (Affymetrix, 701025 Rev. 5) including first and second cDNA generations from oligo-dT and linear *in vitro *transcription using biotinylated ribonucleotides (Enzo, Farmingdale, NY). Samples were hybridized to mgu74av2 arrays at the University of Washington Center for Expression Arrays according to recommended procedures (Affymetrix, 701028 Rev. 3).

Samples were prepared for qRT-PCR using the cDNA archive protocol (Applied Biosystems, Foster City, CA), which uses random hexamers for first strand cDNA synthesis. cDNA samples representing 50 ng total RNA and 2× Universal PCR master mix (Applied Biosystems) were loaded into each port of the 384 well Applied Biosystems Low Density Arrays, which were custom designed for this experiment, and run in an Applied Biosystems 7900 real time PCR instrument according to manufacturer's instructions. Technical and biological replicates were balanced across ports and cards for each of the groups to minimize any effect of loading port position or variability between cards. Each cDNA sample was run in duplicate on 2 different cards.

qRT-PCR data were quantified using SDS 2.1 (Applied Biosystems). For the Low Density Arrays, baseline and threshold were identified automatically by the software and adjusted manually where necessary and the settings were applied to all arrays. Gene expression values were normalized to the 18s endogenous control and corrected for measured efficiency as calculated by a standard curve run on one of the Low Density Arrays [12].

### Selection of genes for qRT-PCR

Genes were selected for qRT-PCR based on an analysis of the entire Affymetrix data set (all 24 individual samples) processed using gcRMA. Chosen genes either exhibited a large average fold-change or highly statistically significant evidence of differential expression as determined by the LIMMA package of Bioconductor [13]. Therefore, selected genes tended to represent genes with large changes for at least one contrast and genes with smaller changes and lower variability.

In detail, we generated gene lists that contained the top 200 candidates for each of the 6 contrasts we study. These gene lists were filtered based on magnitude of signal (average log_2_(signal) >2) and magnitude of change (log_2_(ratio) >0.5) for each contrast. Some genes were chosen based an analysis of additional array data from pools of the RNA samples, choosing the 200 genes with the largest changes for the contrasts listed, and then filtering based on magnitude of signal and magnitude of change as above. 110 genes were selected by these methods. This number was then reduced based on availability of assays on the ABI low density arrays and availability of annotation to yield the 47 genes studied here. The 18s endogenous control is included on the Applied Biosystems Low Density Arrays by default and was used for normalization of the qRT-PCR data as described above.

### Methodologies for array data

All array data were processed in the statistical language 'R' [14] using the "affy" package in Bioconductor [13]. The Bioconductor document available at [15] provides a useful overview of different methods. In our initial "overview" investigation, six different methodologies were applied to array data. Table 2 briefly summarizes the six methodologies in terms of the four stages of data processing (adjustment for background, normalization, use of data from MM probes, and summarizing of data across a probeset). References are provided for background on the methods and they are not described here.

In the follow-up analysis there are three options in each stage of data processing (Table 5), so nominally there would be 81 combinations. However, certain combinations resulted in zero or negative values and could not be further evaluated. See the FollowUpAnalysis.xls. We evaluated 56 combinations in total.

### Contrasts

Our goal in this study is to validate methods for oligonucleotide array data for estimating the relative expression of genes in different RNA samples. However, it would be unwieldy to consider all 276 pairwise comparisons of the 24 RNA samples.

Instead, we consider summary contrasts. Each contrast uses averages among biological replicates.

We give results for six summary contrasts. Three contrasts use the data on all 24 animals (Figure 1): Age (the contrast between the 10 old mice and the 14 young mice), Genotype (the contrast between the 13 MCAT mice and the 11 wild-type mice), and the interaction between age and genotype. We also give results for three simple pairwise group contrasts: YWT vs. OWT (age differences among wild-type mice), YWT vs. YMCAT (genotype differences among young mice), and OWT vs. OMCAT (genotype differences among old mice). We worked with the processed data on the log scale and computed contrasts as differences in per-group averages. The contrasts are interpretable as log-fold changes.

### Quantification of agreement

For each contrast and each method for processing array data, we have a scatterplot. Each of the 47 data points in a scatterplot represents the log-fold-change for one gene for the given contrast as measured by array (vertical axis) and by qRT-PCR (horizontal axis). For a given contrast, the data plotted on the horizontal axis are the same. See Figure 1 for the YWT vs. OWT contrast and the six methods studied in our initial analysis.

We considered several metrics for quantifying agreement within a scatterplot. While it would be ideal if array measurements and qRT-PCR measurements agreed exactly, it is satisfactory for there to be a linear relationship. Therefore, we considered the most important characteristic of these plots to be the overall linear trend. This led us to use Pearson Correlation as a measure of agreement. AdditionalFigures.doc gives results for four other measures of agreement: mean squared error, median absolute error, Canberra distance, and Spearman correlation. However, findings using these four measures were inconclusive.

### Sensitivity analysis

Our measure of agreement, Pearson Correlation, is not robust as it can be disproportionately influenced by individual data points. For example, a gene with a large change for a certain contrast could heavily drive the results for that contrast, but this would be misleading. Therefore, we performed a sensitivity analysis to ensure that our findings were not driven by one or two genes. We systematically removed the data for (1) all single genes and (2) all pairs of genes from the processed datasets, and then re-computed the correlation of the scatterplot with the removed gene(s). We called a gene (or gene-pair) "influential" if the ranking of the six methods changed upon removal of the gene (or gene-pair). For the leave-two-out sensitivity analysis, we only considered gene pairs that were not influential singleton genes.

This sensitivity analysis also takes the place of computing confidence intervals for the correlations we compute. Because the set of genes included in this study was not a random sample, such confidence intervals would not be valid.

## Authors' contributions

LXQ analyzed the qRT-PCR data and performed the overview analysis including the sensitivity analysis. RPB participated in all stages of design and analysis, provided expertise on Bioconductor, and reviewed all code. NJL and FNH conducted all of the RNA assays. NJL additionally provided biological expertise to the project. DEM conducted the exploratory aspect of the study and the follow-up analysis. KFK directed the project and wrote the manuscript. All authors read and approved the final manuscript.

## Supplementary Material

Additional File 1 This is an MS Word document showing the full set of results for the initial analysis. The file includes figures corresponding to Figures 1 and 3 for different contrasts and figures corresponding to Figure 2 for different metrics. Click here for file

Additional File 2 This excel file gives the results of the follow-up analysis. For each combination of sub-methods, the Pearson correlation is given for each contrast. Click here for file

Additional File 3 This MS Word document gives examples from our exploratory analysis seeking associations between probe-level data and agreement between array and qRT-PCR data. Click here for file

## Figures and Tables

**Table 1:** Biological Samples. RNA samples were from an unbalanced 2 × 2 factorial design. The 24 mice were young or old, wild-type or carried the MCAT transgene, which directs overexpression of human catalase to the mitochondrial cellular compartment. Transgene overexpression extends lifespan[16], and thus gene expression differences between MCAT and age-matched wild-type mice would be expected.

	Wild-type	MCAT
Young	N = 6	N = 8
Old	N = 5	N = 5

**Table 2:** Methods under Evaluation. Summary of the six methodologies for oligonucleotide array data that were compared in this study. Details on the methodologies can be found in the references. An asterisk (*) marks components of methods that were studied in the follow-up analysis (see Table 5).

Method	Background Adjustment	Normalization	Mismatch adjustment	Probeset Summary	Reference
MAS5	regional adjustment*	scaling by a constant*	subtract idealized mismatch*	Tukey biweight average*	[17]
gcRMA	by GC content of probe*	quantile normalization*	PM only*	medianpolish* (robust fit of linear model)	[9]
RMA	whole array adjustment	quantile normalization*	PM only*	medianpolish* (robust fit of linear model)	[2]
VSN	none*	variance stabilizing transformation	PM only*	medianpolish* (robust fit of linear model)	[18]
dChip	none*	invariant set*	PM only*	Li-Wong multiplicative	[19]
dChip.mm	none*	invariant set*	subtract mismatch*	model*	

**Table 3:** Results of the leave-one-out sensitivity analysis. For each contrast, an individual gene is listed if its removal produced a change in the ranking of the six methodologies. The third column shows how the ranking of the six methodologies changed upon removal of the gene. Here, M = Mas5, G = gcRMA, R = RMA, V = VSN, D = dChip, D- = dChip.mm. Bold font highlights changes. Note that all changes in rankings, with two exceptions, were transpositions of two adjacent methods or a shuffle of three adjacent methods.

CONTRAST	GENE	RANKING
Age	36	(**M,D-,G**,R,V,D)→(**G,M,D-**,R,V,D)
Genotype	3	(**G,M,D-**,R,V,D)→(**D-,G,M**,R,V,D)
Genotype	12	(**G,M**,D-,**R,V**,D)→(**M,G**,D-,**V,R**,D)
Genotype	26	(G,M,**D-,R**,V,D)→(G,M,**R,D-**,V,D)
Genotype	27	(G,**M,D-**,R,V,D)→(G,**D-,M**,R,V,D)
Interaction	4,19	(M,**D-,G**,R,D,V)→(M,**G,D-**,R,D,V)
Interaction	6,11,24 27,34,37,43,47	(**M,D-**,G,R,D,V)→(**D-,M**,G,R,D,V)
Interaction	8	(M,D-,**G,R**,D,V)→(M,D-,**R,G**,D,V)
Interaction	26	(**M,D-,G**,R,D,V)→(**G,M,D-**,R,D,V)
Interaction	32	(M,D-,G,R,**D,V**)→(M,D-,G,R,**V,D**)
Interaction	12	(**M,D-,G,R,D,V**)→(**G,D-,R,M,V,D**)
YWT-OWT	3	(M,G,D,R,**V,D-**)→(M,G,D,R,**D-,V**)
YWT-OWT	37	(**M,G**,D,R,V,D-)→(**G,M**,D,R,V,D-)
YWT-YMCAT	3	(M,**G,D**,R,V,D-)→(M,**D,G**,R,V,D-)
OWT-OMCAT	2,4,8,15,16,20,27,31,34,35,43,47	(D-,G,V,**M,R**,D)→(D-,G,V,**R,M**,D)
OWT-OMCAT	10,12,17,18,21,36,39,40,45	(D-,G,**V,M**,R,D)→(D-,G,**M,V**,R,D)
OWT-OMCAT	23	(D-,**G,V,M,R**,D)→(D-,**V,G,R,M**,D)
OWT-OMCAT	5,6,13,32,33	(D-,G,**V,M,R**,D)→(D-,G,**R,M,V**,D)
OWT-OMCAT	3	(D-,**G,V,M**,R,D)→(D-,**M,G,V**,R,D)
OWT-OMCAT	26	(**D-,G,V,M,R**,D)→(**G,R,M,V,D-**,D)

**Table 4:** Results of the leave-two-out sensitivity analysis. The table shows that removing gene pairs affected only minor changes in our findings.

CONTRAST	# gene-pairs considered (non-influential singleton genes)	# influential gene-pairs	# of these pairs that produce a single transposition of neighbors	# of these pairs that produce a shuffle of the top-three methods
Age	1035	11	11	0
Genotype	903	3	3	0
Interaction	528	51	43	8
YWT-OWT	990	10	10	0
YWT-YMCAT	1035	14	14	0
OWT-OMCAT	153	2	2	0

**Table 5:** Components of Methods Examined in Follow-Up Analysis. The table gives abbreviations for the methods in Table 2 that are studied in the follow-up analysis. These abbreviations are used in RESULTS and Figures 6–9.

Method	Background Adjustment	Normalization	Mismatch adjustment	Probeset Summary	Reference
MAS5	BA-RA	constant	adjustedMM	TukeyAverage	[17]
gcRMA	BA-GC	quantile	PMonly	medianpolish	[9]
dChip.mm	BA-none	invariantset	subtractMM	Li-Wong	[19]
