# Supplementary material for: Evaluation of methods for oligonucleotide array data via quantitative real-time PCR
Source: BMC Bioinformatics. 2006 Jan 17;7:23. doi: 10.1186/1471-2105-7-23 (PMC1360686; doi:10.1186/1471-2105-7-23)

**Supplement 1. Scatter plots for Affy measures vs. qRT-PCR measures by contrast.**
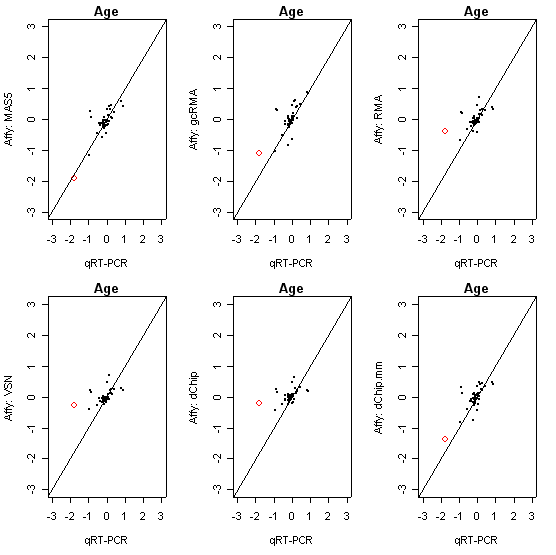

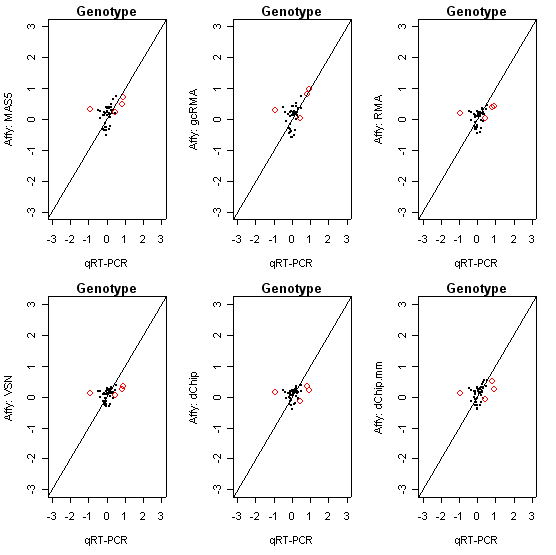

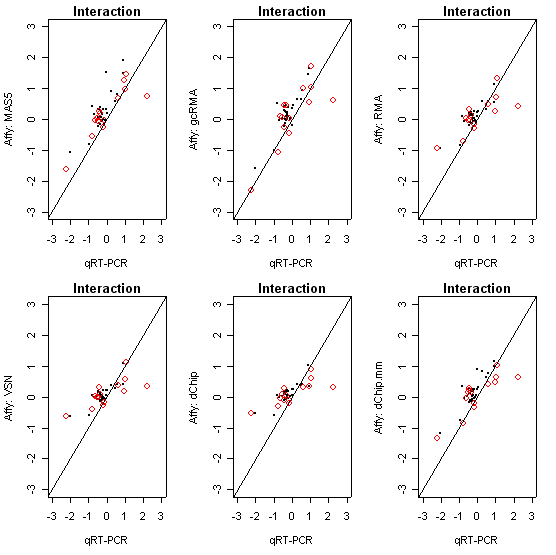

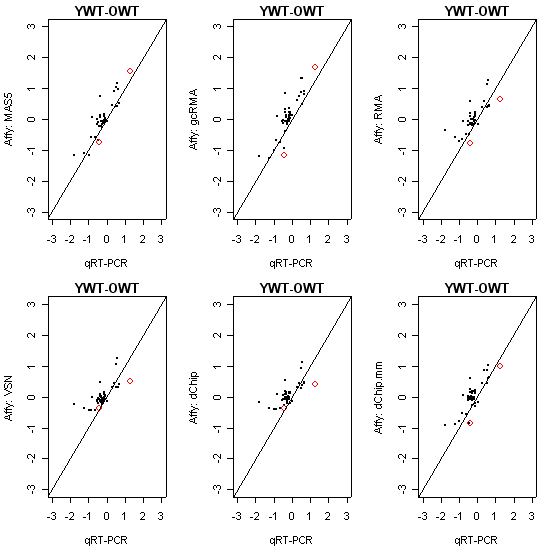

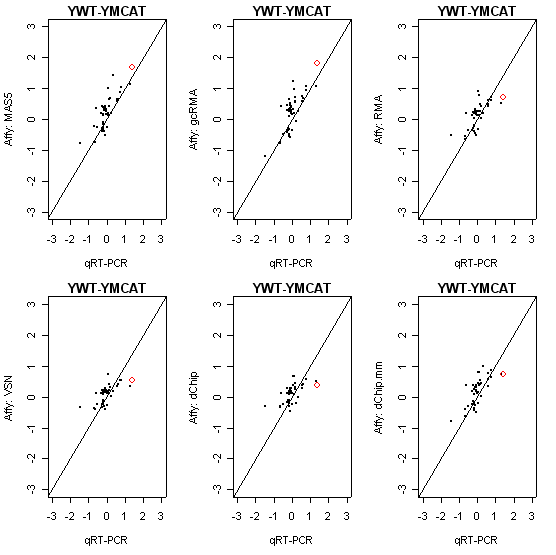

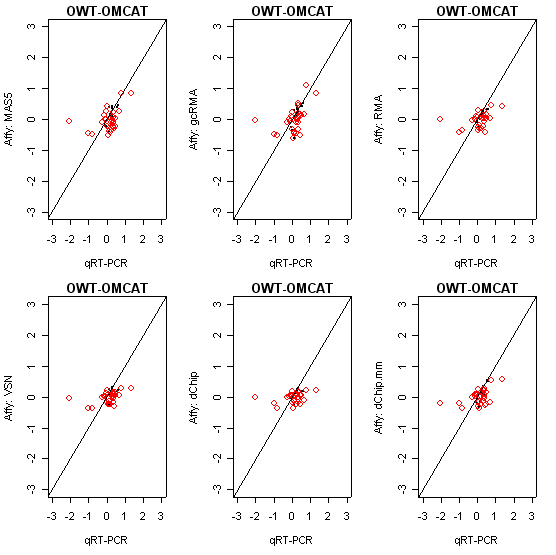


**Supplement 2. Table of Pearson correlations for the six contrasts and the six methods for processing array data. Correlations should be compared within a column. These results are also depicted in Figure 2 in the paper.**

|  | Genotype | Age | Interaction | YWT-OWT | YWT-YMCAT | OWT-OMCAT |
| --- | --- | --- | --- | --- | --- | --- |
| MAS5 | 0.396 | 0.752 | 0.814 | 0.868 | 0.778 | 0.457 |
| gcRMA | 0.422 | 0.691 | 0.809 | 0.861 | 0.766 | 0.473 |
| RMA | 0.323 | 0.550 | 0.797 | 0.756 | 0.679 | 0.456 |
| VSN | 0.315 | 0.468 | 0.771 | 0.710 | 0.642 | 0.458 |
| dChip | 0.223 | 0.434 | 0.777 | 0.700 | 0.600 | 0.370 |
| dChip.mm | 0.365 | 0.710 | 0.812 | 0.822 | 0.721 | 0.498 |

**Supplement 3. Graphical depiction of the performance of different array methodologies using different summary measures on the scatterplots in Supplement 1.**

**
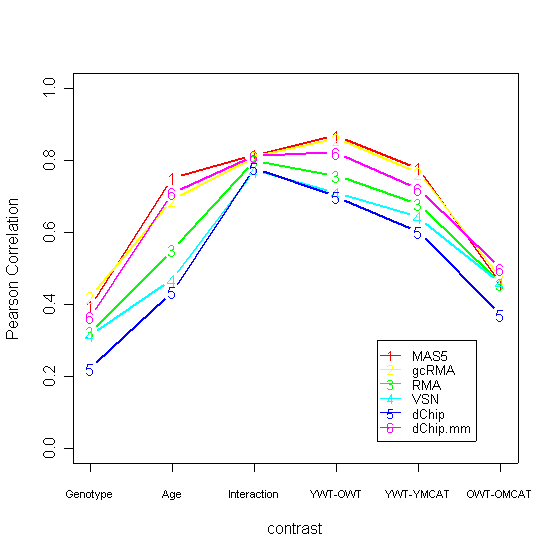
**


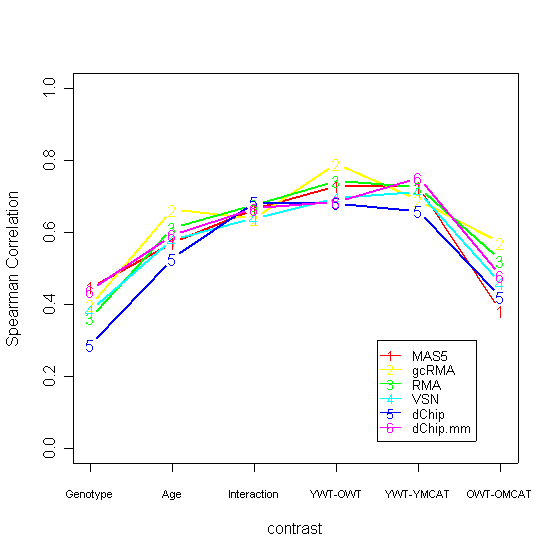
**
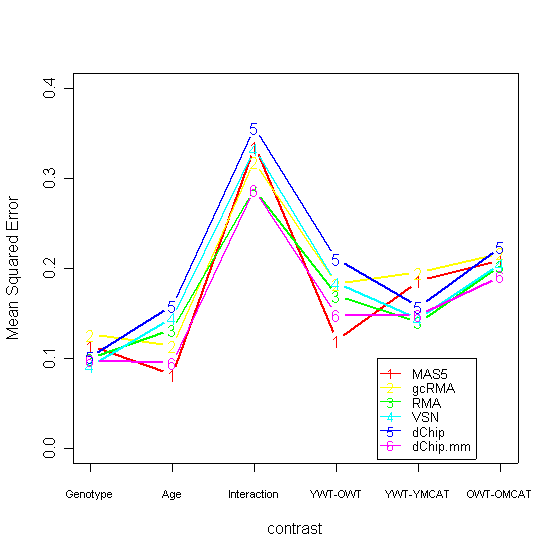
**
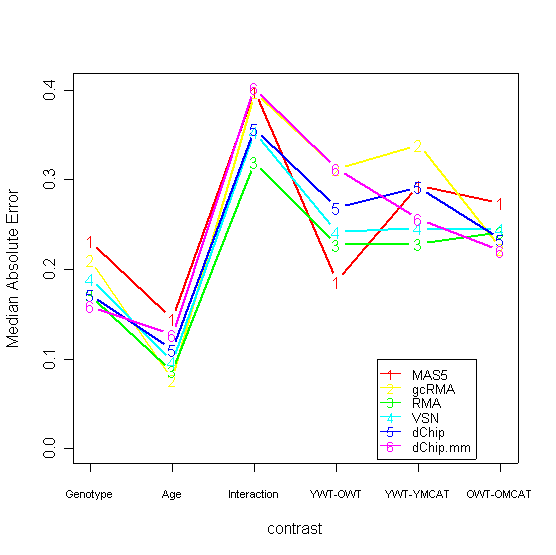

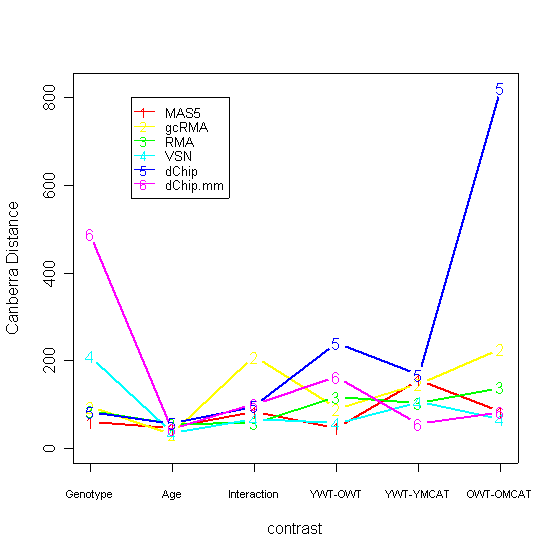


**Supplement 4. Genes selected for qRT-PCR are medium to high intensity in array data**

These plots highlight the genes selected for qRT-PCR as viewed by each contrast against the mean signal intensity. Data were processed with gcRMA for this plot. Selected genes span a large range of average signal intensity with the notable exception of low-intensity genes. Note that the larger fold-changes tend to be for the medium-intensity genes, so these will have more influence on the correlation with qRT-PCR data.


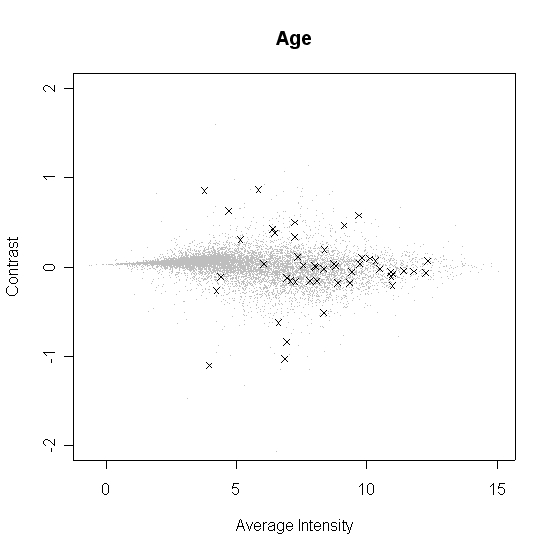


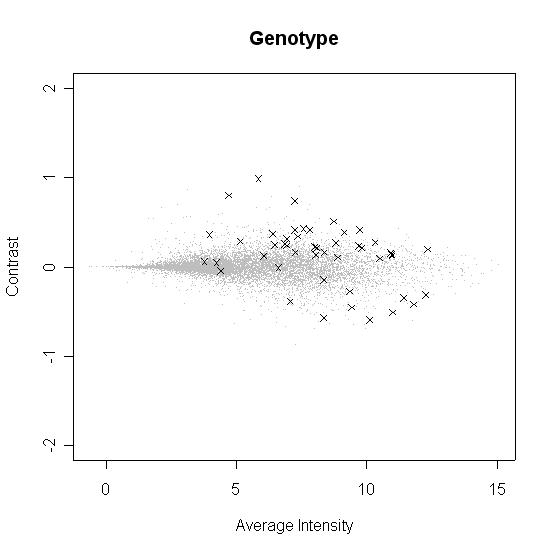


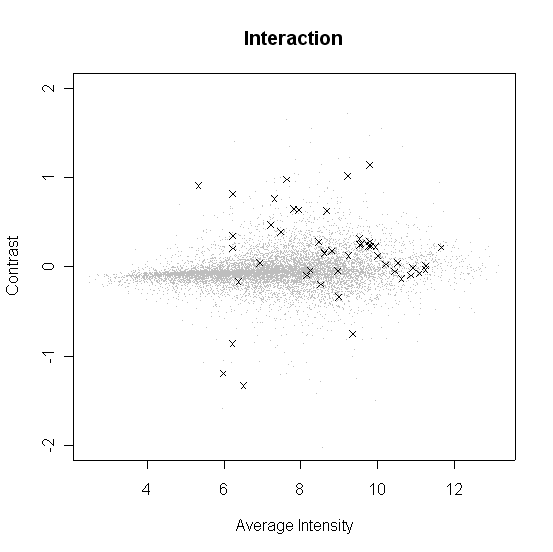

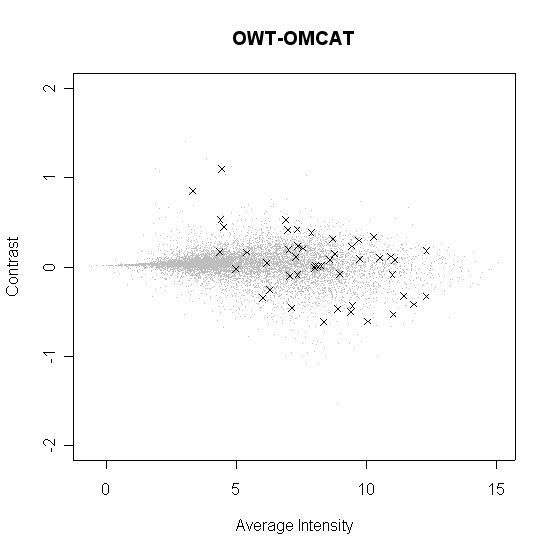


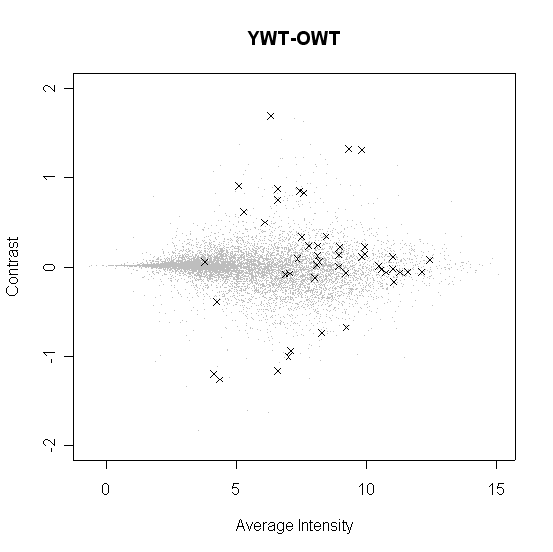


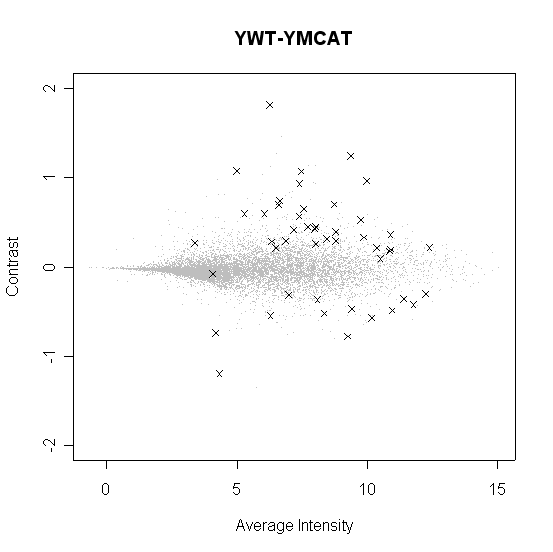

Supplement: Additional File 1 — This is an MS Word document showing the full set of results for the initial analysis. The file includes figures corresponding to Figures 1 and 3 for different contrasts and figures corresponding to Figure 2 for different metrics. [file 1471-2105-7-23-S1.doc]
