# Supplementary figures and images for: Evaluation of methods for oligonucleotide array data via quantitative real-time PCR
Source: BMC Bioinformatics. 2006 Jan 17;7:23. doi: 10.1186/1471-2105-7-23 (PMC1360686; doi:10.1186/1471-2105-7-23)

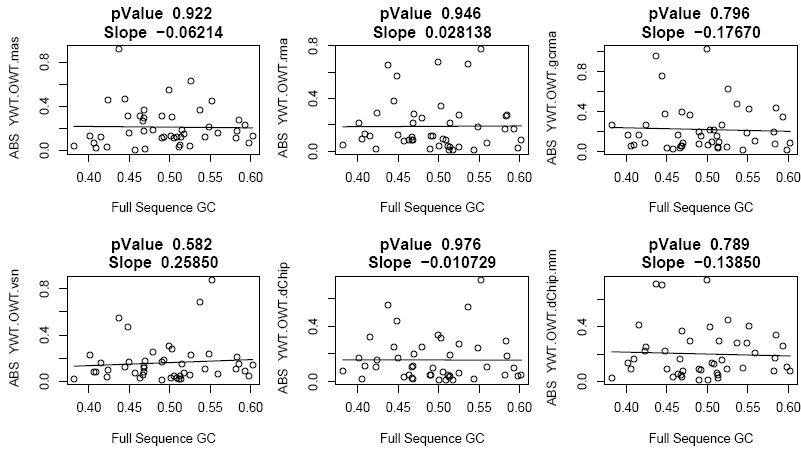


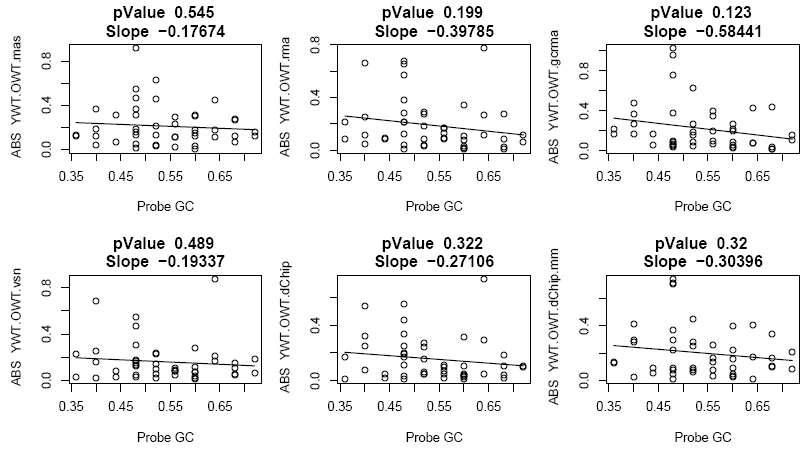


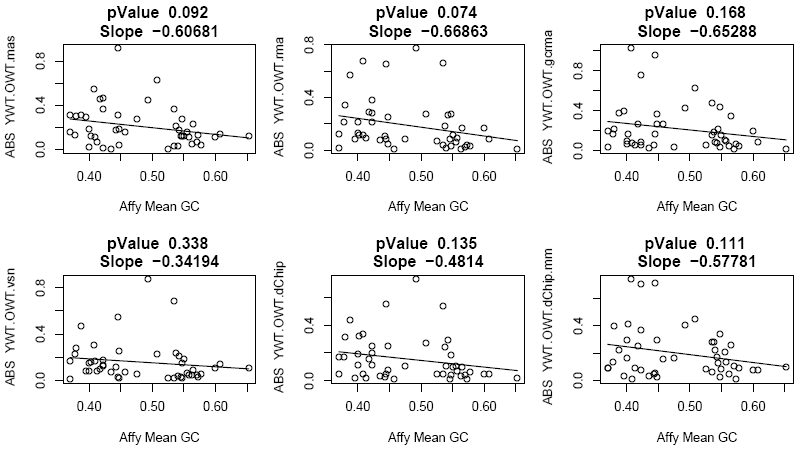


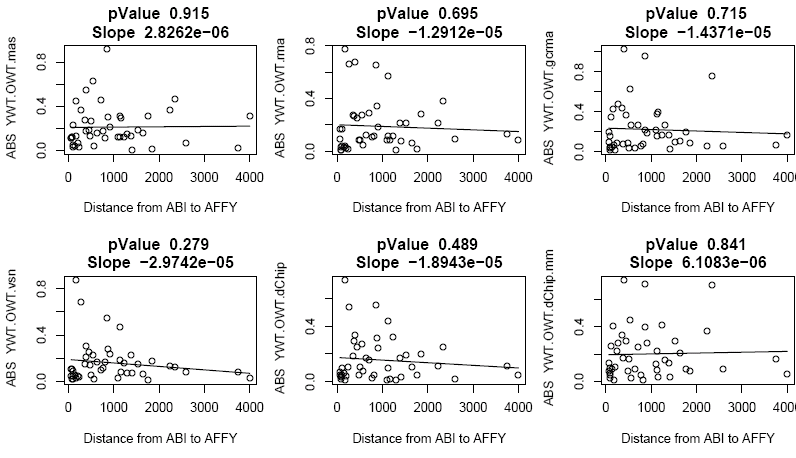

Supplement: Additional File 3 — This MS Word document gives examples from our exploratory analysis seeking associations between probe-level data and agreement between array and qRT-PCR data. [file 1471-2105-7-23-S3.doc]
